# A splice variant of human *Bmal1* acts as a negative regulator of the molecular circadian clock

**DOI:** 10.1038/s12276-018-0187-x

**Published:** 2018-12-06

**Authors:** Jiwon Lee, Eonyoung Park, Ga Hye Kim, Ilmin Kwon, Kyungjin Kim

**Affiliations:** 1 Department of Anatomy and Cell Biology, Sungkyunkwan University School of Medicine, Suwon, 16419 Korea; 2 GenWay Biotech, Inc., 6777 Nancy Ridge Dr, San Diego, CA 92121 USA; 3 Department of Brain and Cognitive Sciences, Daegu Gyeongbuk Institute of Science and Technology (DGIST), Daegu, 42988 Korea

## Abstract

*Bmal1* is one of the key molecules that controls the mammalian molecular clock. In humans, two isoforms of *Bmal1* are generated by alternative RNA splicing. Unlike the extensively studied *hBmal1b*, the canonical form of *Bmal1* in most species, the expression and/or function of another human-specific isoform, *hBmal1a*, are poorly understood. Due to the lack of the N-terminal nuclear localization signal (NLS), hBMAL1a does not enter the nucleus as hBMAL1b does. However, despite the lack of the NLS, hBMAL1a still dimerizes with either hCLOCK or hBMAL1b and thereby promotes cytoplasmic retention or protein degradation, respectively. Consequently, hBMAL1a interferes with hCLOCK:hBMAL1b-induced transcriptional activation and the circadian oscillation of *Period2*. Moreover, when the expression of endogenous *hBmal1a* is aborted by CRISPR/Cas9-mediated knockout, the rhythmic expression of *hPer2* and *hBmal1b* is restored in cultured HeLa cells. Together, these results suggest a role for hBMAL1a as a negative regulator of the mammalian molecular clock.

## Circadian rhythms: Alternative forms of clock protein have opposing effects

An alternative form of a key ‘clock’ protein involved in the maintenance of daily cellular rhythms serves as a negative regulator of the cell’s 24-hour cycle. A team led by Ilmin Kwon from Sungkyunkwan University School of Medicine, Suwon, and Kyungjin Kim from Daegu Gyeongbuk Institute of Science and Technology, both in South Korea, detailed the function of BMAL1a, a lesser-studied variant of the clock protein BMAL1b, in human cells. Whereas BMAL1b enters the nucleus, where it works in concert with another protein called CLOCK to control circadian dynamics, BMAL1a stays in the cytoplasm, where it binds BMAL1b and CLOCK, interfering with their function. Genetically inhibiting BMAL1a helped restore normal rhythmic cycles. Drugs targeting BMAL1a may thus aid in sleep disorders and other circadian-linked health problems.

## Introduction

The regulation of temporal and spatial coordination of gene expression is crucial for living systems. It involves the appropriate activation and/or repression of specific genes^1^. The circadian clock is one of the most well-coordinated biological systems that controls daily oscillations in physiology and behavior. At the molecular level, several core clock genes constitute interlocking feedback loops that drive the rhythmic transcription and translation of the core molecular clock components in the circadian clock system^2,3^. Briefly, heterodimers of CLOCK and BMAL1 (CLOCK:BMAL1) function as transcriptional activators and induce the rhythmic expression of downstream circadian clock genes such as *Periods* (*Pers*) and *Cryptochromes* (*Crys*)^4–6^. Once synthesized in the cytoplasm, Period and Cryptochrome proteins form a complex with Casein kinase I (CKI) and translocate to the nucleus to constitute the negative feedback loop by repressing their own transcriptional activation by CLOCK:BMAL1 heterodimers. Other downstream products of the CLOCK:BMAL1 complex, Rev-erbα and Rorα, constitute additional feedback loops by repressing or activating the transcription of *Bmal1*, respectively^3,7^. These autoregulatory feedback mechanisms of core clock components serve as a fundamental driving force that generates and maintains the circadian rhythm. In addition to the core molecular feedback loops, increasing evidence suggests that post-translational modifications such as phosphorylation, sumoylation, and ubiquitination are closely involved in the fine-tuning of circadian clock control^3,8–12^.

Along with post-translational modifications, alternative RNA splicing is also implicated in the regulation of the molecular circadian clock. For instance, in *Neurospora crassa*, loss of the splicing or mis-splicing of the *White collar-2* gene leads to the production of a nonfunctional protein and subsequent arrhythmicity^13^. In addition, temperature-dependent splicing of another *Neurospora* core clock gene, *Frequency*, is reported to be implicated in the generation of circadian rhythms in *N. crassa*^14,15^. In *Drosophila melanogaster*, flies containing a mutation at the splice site of exon 2 in *Clock* gene exhibit abnormal circadian rhythms^16^. In the *Drosophila Period* gene, the thermosensitive splicing of the intron in the 3′-untranslated region (UTR) is critical for the flies to seasonally adapt to cold temperature^17^.

**Fig. 1:**
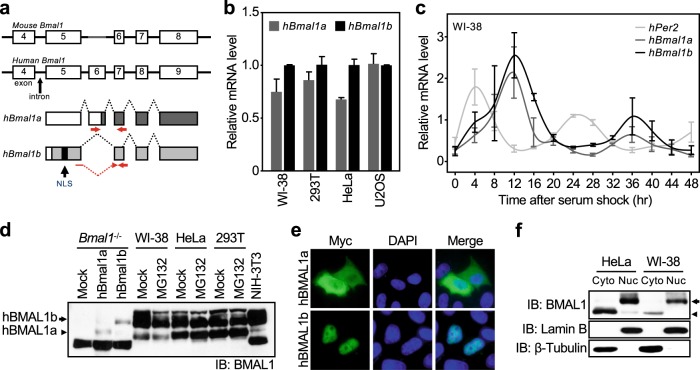
Expression of human Bmal1 splice variants. **a** Schematic of the human and mouse *Bmal1* genes. **b** Expression of *hBmal1a* and *hBmal1b* mRNAs was analyzed by qPCR in various human cell lines. **c** Circadian expression profiles of *hBmal1a* (dark gray line), *hBmal1b* (black line), and *hPer2* mRNAs (light gray line) were analyzed in human fibroblasts, WI-38 cells, by qPCR. **d** Expression of the endogenous hBMAL1a and hBMAL1b proteins was analyzed in several human and mouse cell lines by western blotting. For a size reference, exogenous hBMAL1a or hBMAL1b was expressed in *Bmal1*^−/−^ mouse embryonic fibroblasts (MEFs). **e** The subcellular localization of Myc-tagged hBMAL1a or hBMAL1b was observed under a fluorescence microscope after immunofluorescence staining using an anti-Myc antibody. **f** Expression levels of endogenous hBMAL1a (arrowhead) and hBMAL1b (arrow) in nuclear (Nuc) or cytoplasmic (Cyto) fractions of HeLa or WI-38 cells were investigated by western blotting. Subcellular fractionation was validated by western blotting using anti-β-Tubulin (cytoplasm) or anti-Lamin B (nucleus) antibodies

In mammals, different splice variants of *Bmal1* are generated by alternative RNA splicing in humans, mice, and rats^18–20^. Based on an analysis of the sequences of different *Bmal1* variants produced in three different species, we noticed that the human *Bmal1a* isoform (*hBmal1a*) was the only variant in which the N-terminal 43 amino acids are missing (Supplementary Fig. S1a). When exon 6 of the human *Bmal1* gene is removed by RNA splicing, the canonical isoform *hBmal1b* is generated from the translational start signal located in exon 5 (Fig. 1a). However, when exon 6 is retained, the synthesis of *hBmal1b* is terminated by the premature stop codon in exon 6 and another translation begins at the alternative initiation codon in the same exon to produce *hBmal1a* without the N-terminal region (Fig. 1a).

We began to focus on *hBmal1a* because the nuclear localization signal (NLS) resides in the N-terminal region that is absent in *hBmal1a* (Supplementary Fig. S1a). Moreover, in the previous studies, it was reported that the NLS of *Bmal1* is required for the nuclear localization of BMAL1 and its binding partner, CLOCK, and subsequent transcriptional activation of core clock genes by CLOCK:BMAL1 heterodimers in the nucleus^21,22^. The alternative RNA splicing of human *Bmal1* and production of *hBmal1a* was first reported more than 20 years ago^18^. *Bmal1* is a master transcriptional regulator of the mammalian molecular clock. However, little information is available about the functional implications of different kinds of *Bmal1* variants in the regulation of the mammalian circadian clock. Here, we investigate for the first time the functional role of the human-specific *Bmal1* variant, hBMAL1a, in the control of the molecular circadian clock.

## Materials and methods

### Cell culture and transient transfection

WI-38, HEK293T, HeLa, and NIH-3T3 cells were grown in Dulbecco's modification of Eagle medium supplemented with 10% fetal bovine serum (Thermo Fisher Scientific, Waltham, MA, USA) and 1% penicillin–streptomycin (Thermo Fisher Scientific) at 37 °C. Transient transfection was performed using Lipofectamine 2000 reagent (Thermo Fisher Scientific), according to the manufacturer’s protocol.

### RT-PCR and qPCR

Total RNA was isolated using Trizol (Thermo Fisher Scientific), according to the manufacturer’s protocol. RNA samples were treated with Turbo DNase (Thermo Fisher Scientific) for 30 min at 37 °C. The RNA was reverse transcribed using the Superscript IV first-strand synthesis system (Thermo Fisher Scientific), according to the manufacturer’s protocol. The resulting cDNAs were subjected to PCR amplification using the following primers: hBmal1b-F (5′-GCTCCACTGACTACCAAGAAAG-3′), hBmal1a-F (5′-GCTCCACTGACTACCATTCACA-3′), and hBmal1-R (5′-GCTGAACAGCCATCCTTAGC-3′).

For qPCR, 1 μg of DNase-treated RNA was reverse transcribed using random hexamers and Superscript IV reverse transcriptase. The level of each mRNA was normalized to *Gapdh* as an internal control, and the result was plotted as a normalized fold-change versus the control. Primers used for qPCR were: *Gapdh-*F (5′-TGCACCACCAACTGCTTAGC-3′), *Gapdh*-R (5′-GCAGTGATGGCATGGACTGT-3′), *hBmal1a-*F (5′-CAAAGATGACCCTCATGGAAG-3′), *hBmal1a-*R (5′-CTTCCATGAGGGTCATCTTTG-3′), *hBmal1b-*F (5′-TCCACTGACTACCAAGAAAGCA-3′), *hBmal1b-*R (5′-ACTGTGAGCTTCCCTTGCAT-3′), *hPer2-*F (5′-CCAGTAACCCCACCAAGGAG-3′), and *hPer2-*R (5′-ACTGCTCATGTCCACATCTTCC-3′).

### Nucleocytoplasmic fractionation

HeLa, WI-38, or Myc-hBmal1-expressing HeLa cells were harvested in buffer A (50 mM HEPES, pH 7.4, 1.5 mM MgCl_2_, and 10 mM KCl) and incubated on ice for 15 min. After centrifugation at 3,000 rpm for 10 min at 4 °C, the supernatant was transferred to a new tube. For cytoplasmic fraction, the supernatant was centrifuged at 12,000 rpm for 10 min at 4 °C and added to 4× sample buffer. For nuclear fraction, the pellet was resuspended in buffer C (50 mM HEPES, pH 7.4, 1.5 mM MgCl_2_, 10 mM KCl, and 450 mM NaCl) and incubated on ice for 30 min. Samples were centrifuged at 12,000 rpm for 10 min at 4 °C and the supernatants were collected. Fractionation was confirmed by examining the expression of Lamin B and β-Tubulin in the nuclear and the cytoplasmic fractions, respectively.

### Immunoprecipitation and western blotting

HeLa cells were transfected with Flag-tagged hBmal1b and an increasing amount of Myc-tagged hBmal1a. After an incubation for 48 h, cells were lysed with 500 μl of IP buffer [50 mM HEPES, pH 7.4, 150 mM NaCl, 1% NP-40, 1 mM EDTA, 1 mM EGTA, 1 mM PMSF, 0.5% Na-deoxycholate and a protease inhibitor cocktail (Sigma-Aldrich, St. Louis, MO, USA)] and centrifuged at 12,000 rpm for 20 min at 4 °C. After quantification by the Bradford assay, lysates were incubated with 2 μg of anti-Flag antibody for 2 h at 4 °C and then incubated with a protein G-sepharose bead slurry for 1 h at 4 °C. The final immune complexes were eluted with 2× sodium dodecyl sulfate (SDS) sample buffer and analyzed by western blotting.

For western blotting, protein samples were separated on 6% SDS-polyacrylamide gels and transferred to PVDF membranes. Western blotting was performed with anti-Myc (9E10; Santa Cruz Biotechnology, Dallas, TX, USA), anti-Flag (M2; Sigma-Aldrich), anti-CLOCK (S19; Santa Cruz Biotechnology), anti-BMAL1 (Novus Biologicals, Littleton, CO, USA), or anti-BMAL1^22^ antibodies and HRP-conjugated secondary antibodies for enhanced chemiluminescence detection.

### Bimolecular fluorescence complementation assay and immunofluorescence

The bimolecular fluorescence complementation (BiFC) assay was performed as described elsewhere^23^. Briefly, the N-terminal 173 amino acid residues of the YFP (YN) and the C-terminal amino acid residues 173–238 of YFP (YC) were used to prepare fusion gene constructs for use in the BiFC assay. For the dual-color BiFC assay^24^, the partial sequences of CFP encoding amino acid residues 1–173 (CN) or amino acid residues 173–238 (CC) were used to construct the fusion genes. Fusion gene plasmids were transfected into HeLa cells. Twenty-four hours after transfection, cells were fixed with 4% paraformaldehyde in phosphate-buffered saline (PBS). After 1 h of blocking with 5% donkey serum and 0.3% Triton X-100 in PBS at room temperature, fixed cells were incubated with the indicated primary antibodies overnight at 4 °C. Cells were then washed with PBS and incubated with fluorescent dye-conjugated secondary antibodies for 1 h at room temperature. Samples were analyzed by confocal microscopy (Nikon, Tokyo, Japan).

### Chromatin immunoprecipitation assay

Myc-tagged hBmal1b was transfected with an increasing amount of Flag-hBmal1a into HeLa cells. Forty-eight hours after transfection, cells were fixed with 1% paraformaldehyde for 10 min at 37 °C. Cells were incubated with Lysis buffer (50 mM Tris-HCl, pH 8.0, 150 mM NaCl, 2% Triton X-100, 10 mM EDTA, and 1% SDS) for 10 min at 4 °C and then sonicated to shear the chromatin. After centrifugation at 12,000 rpm for 10 min at 4 °C, the supernatant was transferred into new tubes and was diluted 10-fold with Dilution buffer (50 mM Tris-HCl, pH 8.0, 150 mM NaCl, 0.1% Triton X-100, 10 mM EDTA, and 1 mM PMSF). Each cross-linked sample was immunoprecipitated with 2 μg of anti-CLOCK (S19; Santa Cruz Biotechnology) or anti-Myc (9E10; Santa Cruz Biotechnology) antibodies at 4 °C overnight. Then, protein G-sepharose beads were added to each tube and incubated for 2 h at 4 °C. The pellet was washed with low salt buffer (20 mM Tris-HCl, pH 8.0, 150 mM NaCl, 1% Triton X-100, 2 mM EDTA, and 0.1% SDS), high salt buffer (20 mM Tris-HCl, pH 8.0, 500 mM NaCl, 1% Triton X-100, 2 mM EDTA, and 0.1% SDS), LiCl buffer (10 mM Tris-HCl, pH 8.0, 250 mM LiCl, 1% NP-40, 1 mM EDTA, and 1% Na-deoxycholate), and TE buffer (10 mM Tris-HCl, pH 8.0, and 1 mM EDTA). Samples were eluted with Elution buffer (1% SDS and 0.1 M NaHCO_3_) and incubated with 0.2 M NaCl for 6 h at 65 °C to reverse the cross-linking. Then, DNA was purified by phenol–chloroform extraction and ethanol precipitation. For PCR amplification of the proximal *hPer1* E-box, the following primers were used: F (5′-GGAGGAAAGTACTAGACACCACGTA-3′) and R (5′-CTCCCAACCTTCGTTCTACATAAT-3′).

### Real-time monitoring of clock gene promoter expression

For the real-time monitoring of circadian rhythmicity, NIH-3T3 cells were transfected with the *hPer2*-promoter conjugated to destabilized firefly luciferase together with hBmal1a and/or hBmal1b, as indicated. When transfected cells reached confluence, 1 μM dexamethasone (Dex; Sigma-Aldrich) was applied for 2 h to induce and synchronize the circadian rhythms. Real-time luciferase activity was measured using Kronos-Dio (Atto, Tokyo, Japan) for 72 h.

### Generation of CRISPR/Cas9-mediated *hBmal1a* knockout cells

Genome editing for *hBmal1a* knockout was performed as previously described^25^. The sgRNA constructs designed to skip exon 6 were inserted into the pSpCas9(BB)-2A-Puro (PX459) plasmid. The guide sequences were: sgRNA*-*i5 (5′-ATTTGGGTAAGATTCCACGC-3′) and sgRNA*-*i6 (5′-TTAGCGCTTGCTCAAGACA A-3′).

After transfection of sgRNA constructs, HeLa cells were incubated for 72 h and genomic DNA was extracted using the QuickExtract DNA extraction solution. The genomic region containing exon 6 of *hBmal1* gene was amplified using the following primers: F (5′-TGGAAGGAATGAGTGGAGGT-3′) and R (5′-GGCCAGGTGATAATCTCAGG-3′)

Heteroduplex DNA was obtained by the denaturation and renaturation of genomic PCR samples and then quantified using the T7 Endonucelase1 (T7E1) assay (New England Biolabs, Ipswich, MA, USA).

HeLa cells stably expressing sgRNA-i5 and/or sgRNA-i6 were selected with puromycin. Genomic PCR and DNA sequencing were performed to detect the specific cells in which exon 6 of *Bmal1* was deleted. RNA was isolated from HeLa cells or sgRNA-expressing cells and reverse transcribed using the Superscript IV first-strand synthesis system (Thermo Fisher Scientific) to confirm the expression of *hBmal1a* mRNA. RT-PCR was performed using the following primer sequences: *hBmal1-*exon4-F (5′-TGAAAATCGCTTTGAGGTGA-3′), *hBmal1a-*exon6-F  (5′-GGTCAGATGCCCACTAGGAG-3′), *hBmal1b-*exon5&7-F (5′-GCTCCACTGACTACCAAGAAAG-3′), and *hBmal1-*exon9-R (5′- GCTGAACAGCCATCCTTAGC-3′).

### Statistical analysis

The data are presented as the means ± SE. The statistical significance of differences between groups was determined using one-way ANOVA. *P* < 0.01 was regarded as a significant difference.

## Results

### Expression of *hBmal1a* in human cell lines

First, the expression of *hBmal1a* and *hBmal1b* mRNAs was analyzed with quantitative PCR (qPCR) using the designated primers (Fig. 1a). As shown in Fig. 1b, a significant amount of *hBmal1a* mRNA was detected in different human cell lines. However, the expression of a *Bmal1* isoform corresponding to *hBmal1a* was not detected in the mouse cell line NIH-3T3 and different mouse tissues (Supplementary Fig. S1b). The expression of a substantial amount of *hBmal1a* mRNA was also validated by RT-PCR (Supplementary Fig. S1c and d). Next, we examined the circadian expression profiles of *hBmal1a*, *hBmal1b*, and *hPer2*, a negative regulator of the molecular clock in cultured WI-38 human fibroblasts. Upon application of 50% horse serum for 2 h, *hPer2* and *hBmal1b* mRNAs exhibited an anti-phasic circadian expression pattern for up to 48 h, consistent with previous reports^26–28^ (Fig. 1c). As shown in Fig. 1c, the expression profile of *hBmal1a* mRNA was similar to *hBmal1b*.

Total lysates obtained from the human cell lines WI-38, HeLa, and HEK293T were subjected to western blotting using an anti-BMAL1 antibody to analyze the expression of endogenous hBMAL1a and hBMAL1b^22^. As shown in Fig. 1d, two distinct BMAL1 bands were detected in all three cell lines. Considering the difference in the sizes of hBMAL1b (68.8 kDa) and hBMAL1a (64.2 kDa), the upper (arrow) and lower (arrowhead) bands were predicted to represent hBMAL1b and hBMAL1a, respectively (Fig. 1d). Moreover, the lower band was not detected in the lysates of NIH-3T3 cells (Fig. 1d). In an effort to validate whether the lower band indeed corresponded to hBMAL1a, plasmids expressing hBmal1a or hBmal1b were transfected into *Bmal1* knockout mouse embryonic fibroblasts (MEF^*Bmal1−/−*^) in which no BMAL1 band was detected in western blotting (Fig. 1d, lanes 1–3). The difference in the sizes of exogenously expressed hBMAL1a and hBMAL1b was similar to the difference in the size of the two endogenous protein bands detected using the BMAL1 antibody (Fig. 1d). A proteasome inhibitor, MG132, had no effect on the intensity of the two BMAL1 bands, suggesting that the lower band is not a degraded form of hBMAL1b (Fig. 1d). BMAL1 was detected as hypo- or hyper-phosphorylated form on western blotting in the previous study^26^. To test the possibility that the lower BMAL1 band is the hypo- or unphosphorylated form of the hBMAL1b, a pull-down assay was performed following the application of phosphatases. As shown in Supplementary Fig. S2a–c, phosphatases had no effect on the mobility of both endogenous and exogenously expressed BMAL1 protein bands.

Due to the absence of the NLS, Myc-tagged hBMAL1a predominantly localized in the cytoplasm, while Myc-tagged hBMAL1b entered nuclei (Fig. 1e). In the western blotting, exogenously expressed Flag-tagged hBMAL1a was detected in both cytoplasmic and nuclear fractions, while Myc-tagged hBMAL1b was predominantly detected in the nuclear lysates (Supplementary Fig. S2d). When different human cell lysates were subjected to western blotting with an anti-BMAL1 antibody after nucleocytoplasmic fractionation, the lower BMAL1a band was predominantly detected in the cytoplasmic fraction, while the upper band was predominantly detected in the nuclear fraction (Fig. 1f).

### hBMAL1a-mediated cytoplasmic retention of hCLOCK

**Fig. 2:**
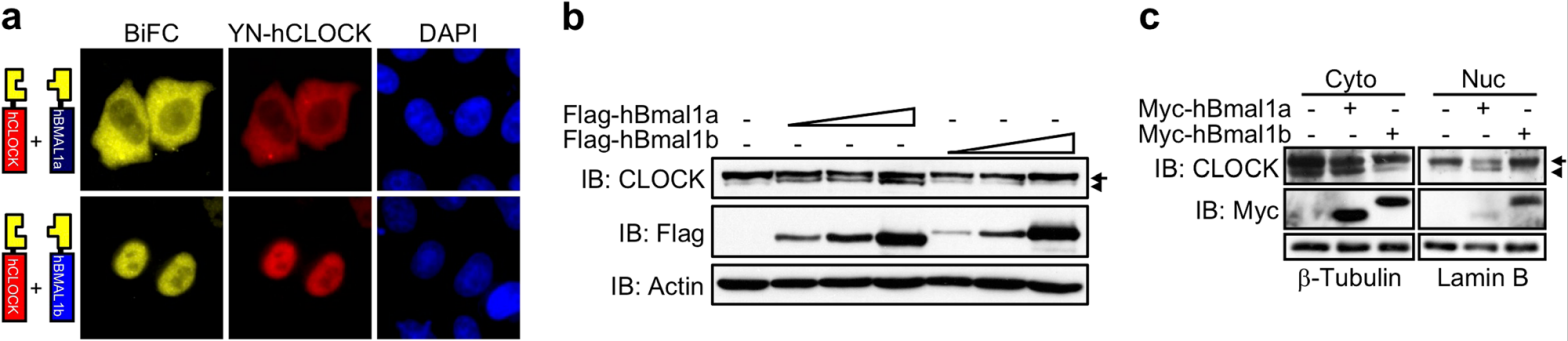
Cytoplasmic retention of hCLOCK by hBMAL1a. **a** Direct interactions between hBMAL1 isoforms and hCLOCK were visualized by the BiFC assay. YN-hClock was co-transfected with YC-hBmal1a or YC-hBmal1b into HeLa cells. The subcellular localization of hCLOCK was detected by immunofluorescence staining using an anti-CLOCK antibody. **b** The expression of the endogenous hCLOCK was determined by western blotting in the presence of different levels of either Flag-hBMAL1a or hBMAL1b. **c** HeLa cells transfected with either Myc-hBmal1a or Myc-hBmal1b were subjected to nucleocytoplasmic fractionation. The level of hCLOCK in each fraction was analyzed by western blotting using an anti-CLOCK antibody

Although the NLS is missing, hBMAL1a still possesses intact basic helix–loop–helix (bHLH) and PER-ARNT-SIM (PAS) domains that are required for the interaction with its binding partner hCLOCK^22^. Additionally, nuclear entry of the CLOCK:BMAL1 complex is dependent on the NLS of BMAL1^22^. Thus, hBMAL1a is presumed to form a complex with hCLOCK in the cytoplasm and thereby prevent hCLOCK from entering the nucleus. To visualize this possibility, the direct interaction between the two different hBMAL1 isoforms and hCLOCK was analyzed by BiFC assay^9,23^. In consistent with the previous report^22^ and as expected, the YFP signal representing the direct interaction between YFP N-terminus (YN)-tagged hCLOCK (YN-hCLOCK) and YFP C-terminus (YC)-tagged hBMAL1b (YC-hBMAL1b) was detected in the nuclei (Fig. 2a, bottom panels), while the YFP signal of YN-hCLOCK and YC-hBMAL1a complex was predominant in the cytoplasm (Fig. 2a, top panels).

When analyzed by western blotting, two differently sized hCLOCK proteins were detected (Fig. 2b, first lane). This finding is consistent with the previous report in which the electrophoretic mobility of CLOCK was altered by phosphorylation upon serum application to cultured cells^29,30^. Interestingly, the intensity of the fast-migrating lower band of hCLOCK was decreased by hBMAL1b in a dose-dependent manner (Fig. 2b, lanes 5–7), while the amount of the lower hCLOCK band was increased in the presence of hBMAL1a (Fig. 2b, lanes 2–4). In the pull down assay, Myc-tagged hBMAL1a or hBMAL1b predominantly coprecipitated with the lower or upper bands of hCLOCK, respectively (Supplementary Fig. S3). To further analyze the effect of hBMAL1 isoforms on hCLOCK, nuclear or cytoplasmic fractions were subjected to western blotting in the presence of hBMAL1a or hBMAL1b. First, as shown in Fig. 2c, lanes 1 and 4, in samples without any exogenous hBMAL1 proteins, the upper band was the dominant form of hCLOCK in the nucleus, while both upper and lower bands were detected in the cytoplasm. In the presence of overexpressed hBMAL1a, the amount of upper band in the nucleus decreased, while no apparent change was observed in the cytoplasm (Fig. 2c, lanes 2 and 5). On the other hand, the amount of the lower hCLOCK band in the cytoplasm decreased, while the amount of the upper hCLOCK band in the nucleus increased upon overexpression of hBMAL1b (Fig. 2c, lanes 3 and 6). Based on these data, hBMAL1b promotes the nuclear entry of hCLOCK, while hBMAL1a prevents hCLOCK from entering the nucleus.

### hBMAL1a promotes the proteolysis of hBMAL1b

**Fig. 3:**
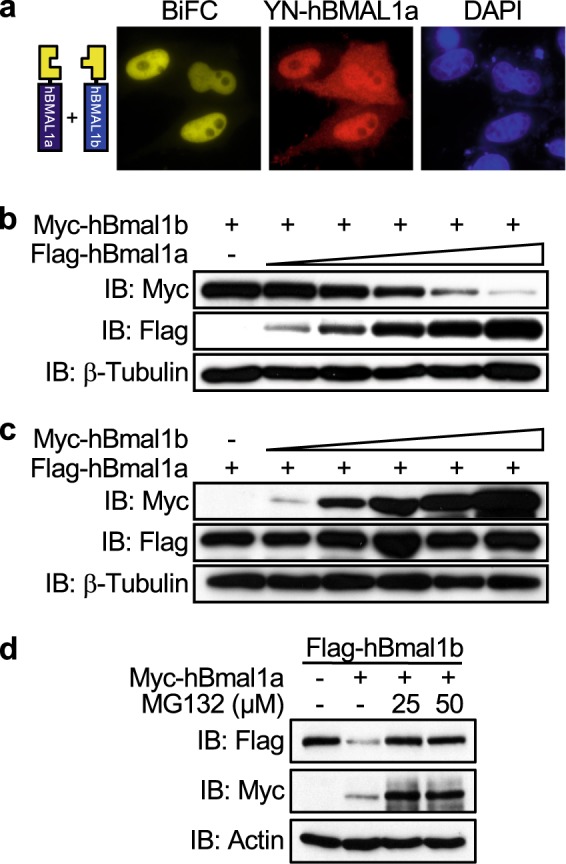
hBMAL1a promotes the proteolysis of hBMAL1b. **a** The direct interaction between hBMAL1a and hBMAL1b was visualized by the BiFC assay using YN-Flag-fused hBmal1a and YC-fused hBmal1b. **b**, **c** Fixed amounts of Myc-hBmal1b or Flag-hBmal1a were co-transfected into HeLa cells with increasing amounts of Flag-hBmal1a or Myc-hBmal1b, respectively. Levels of Myc-hBMAL1a or Flag-hBMAL1b were detected by western blotting. **d** Flag-hBmal1b was transfected alone or together with Myc-hBmal1a into HeLa cells. After an overnight incubation, a potent protease inhibitor, MG132, was applied to the transfected cells as indicated. After 5 h incubation, levels of hBMAL1 isoforms were analyzed by western blotting

Theoretically, hBMAL1a, which has no NLS, should be predominantly retained in the cytoplasm. However, as shown in Fig. 1e and Supplementary Fig. S2d, a significant amount of hBMAL1a was still detected in the nucleus. Thus, hBMAL1a might be transported into the nucleus by another binding partner. In the previous study, it was reported that the recombinant BMAL1 protein forms BMAL1:BMAL1 homodimer complex in vitro^31^. Therefore, we hypothesized that hBMAL1a might enter the nucleus in the form of homodimer with hBMAL1b. To test this hypothesis, YN-hBmal1a and YC-hBmal1b were co-expressed in cultured cell lines. As expected, YFP signal representing the hBMAL1a:hBMAL1b complex was mainly detected in the nuclei (Fig. 3a, left panel), while the immunofluorescence signal for YN-hBMAL1a was detected in both the nuclei and cytoplasm (Fig. 3a, middle panel).

In the course of studying the effects of hBMAL1a:hBMAL1b homodimer formation, hBMAL1a was observed to promote the proteolysis of hBMAL1b in a dose-dependent manner (Fig. 3b; Supplementary Fig. S4). However, hBMAL1b had no effect on the stability of hBMAL1a protein (Fig. 3c). Moreover, hBMAL1a-mediated degradation of hBMAL1b was reversed upon application of MG132, a potent inhibitor of the 26S proteasome (Fig. 3d). These data are consistent with the previous report showing that the splice variants of human luteinizing hormone receptor control the stability of the other variants through a direct interaction^32^. Taken together, hBMAL1a directly binds hBMAL1b and thereby induces the proteolysis of hBMAL1b.

### hBMAL1a interferes with the formation of hCLOCK:hBMAL1b heterodimers

**Fig. 4:**
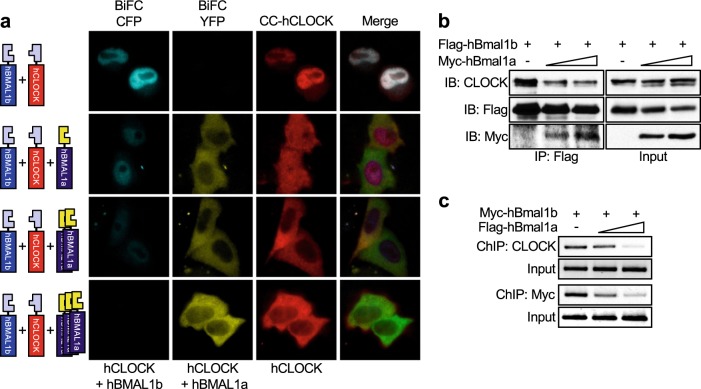
The effect of hBMAL1a on the formation of the hBMAL1b and hCLOCK heterodimer complex. **a** CN-hBmal1b and CC-hClock were co-transfected with increasing amounts of YN-hBmal1a to visualize the effect of hBMAL1a on the heterodimerization of hCLOCK and hBMAL1b. Twenty-four hours after transfection, the signals for hCLOCK:hBMAL1b complex (CFP) or hCLOCK:hBMAL1a complex (YFP) were detected under a fluorescence microscope. The subcellular localization of CC-hCLOCK was also detected by immunofluorescence staining using an anti-CLOCK antibody. **b** The effect of hBMAL1a on the formation of the hCLOCK:hBMAL1b complex was further investigated by an immunoprecipitation assay. **c** The effect of hBMAL1a on the recruitment of either hBMAL1b or hCLOCK to the E-box element of the *hPer1* promoter was analyzed by chromatin immunoprecipitation (ChIP) assay

Having observed that hBMAL1a formed complexes with either hBMAL1b or hCLOCK, we hypothesized that hBMAL1a might compete with hBMAL1b for heterodimer formation with hCLOCK. To test this hypothesis, a dual-color BiFC assay was performed using N-terminal CFP (CN)-tagged hBmal1b (CN-hBmal1b), C-terminal CFP (CC)-tagged hClock (CC-hClock), and YN-hBmal1a^9,23^. In the absence of YN-hBMAL1a, CFP signals representing the hCLOCK:hBMAL1b complex were detected in the nuclei (Fig. 4a, top row). However, nuclear CFP signals were diminished while cytoplasmic YFP signals representing hCLOCK:hBMAL1a were increased following the co-expression of YN-hBMAL1a in a dose-dependent manner (Fig. 4a, first and second columns). Simultaneously, cytoplasmic retention of hCLOCK was also dose-dependently increased by YN-hBMAL1a (Fig. 4a, third column). Consistent with these findings, pull down of endogenous hCLOCK by Myc-tagged hBMAL1b was dose-dependently decreased by the co-expression of Flag-tagged hBMAL1a (Fig. 4b).

For the activation of downstream core clock genes, heterodimers of CLOCK:BMAL1 must be recruited to the E-box cis-elements in the promoter region of downstream genes^30^. A chromatin immunoprecipitation assay was performed to determine whether hBMAL1a inhibits the recruitment of either hCLOCK or hBMAL1b to the E-box element. In the absence of hBMAL1a, both hCLOCK and exogenously expressed Myc-hBMAL1b were effectively recruited to the proximal E-box of the *Period1* promoter (Fig. 4c, first lane). However, the E-box recruitment of hCLOCK or Myc-hBMAL1b was decreased by Flag-hBMAL1a in a dose-dependent manner (Fig. 4c, second and third lanes). Taken together, these data suggest the evidence that hBMAL1a interferes with the formation and subsequent E-box recruitment of hCLOCK:hBMAL1b heterodimers.

### hBMAL1a functions as a negative regulator of the molecular clock

**Fig. 5:**
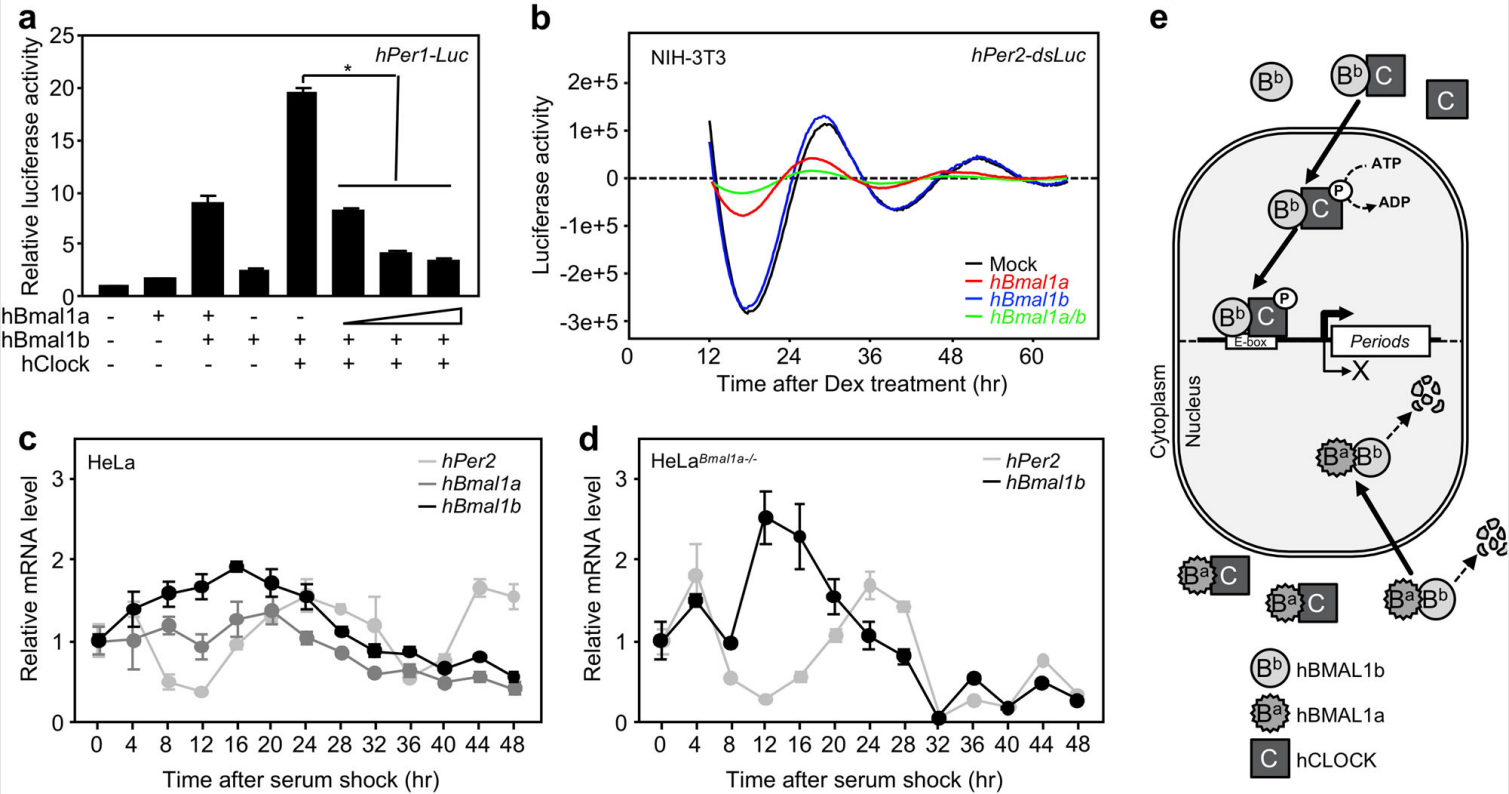
Dominant-negative effect of hBMAL1a on the regulation of the molecular circadian clock. **a** The activation of *hPer1* promoter linked to firefly luciferase was assayed in HEK293T cells transfected with the indicated plasmid DNAs. The relative luciferase activities are shown as the means ± standard errors (S.E.) (*P < 0.01). **b** The effect of hBMAL1a and/or hBMAL1b on the rhythmic expression profile of the *hPer2* promoter linked to destabilized firefly luciferase (*dsLuc*) was measured in NIH-3T3 cells for 72 h using Kronos-Dio (Atto). **c**, **d** Circadian expression levels of *hBmal1a*, *hBmal1b*, and *hPer2* mRNAs in HeLa or HeLa^*Bmal1a−/−*^ cells were analyzed. Following the application of 50% horse serum for 2 h, relative mRNA levels were determined by qPCR for 48 h. **e** A model for the dominant-negative function of hBMAL1a. In the cytoplasm, hBMAL1a interferes with the formation of hCLOCK:hBMAL1b heterodimer complex by binding hCLOCK or hBMAL1b proteins. Through this direct interaction, hBMAL1a prevents hCLOCK phosphorylation and nuclear translocation, which are closely related to the transcriptional activity of hCLOCK. The hBMAL1a protein also promotes the proteolysis of hBMAL1b through a direct interaction. Consequently, hBMAL1a functions as a dominant-negative regulator in the control of the molecular circadian clock

Knowing that hBMAL1a interferes with the formation of the functional transcriptional activator complex between hCLOCK and hBMAL1b, we evaluated the effect of hBMAL1a on the transcriptional activation mediated by hCLOCK:hBMAL1b heterodimers. In a reporter assay using human *Period1* (*hPer1*) promoter conjugated to the firefly *Luciferase* (*hPer1-Luc*), hBMAL1b and hCLOCK-induced transcriptional activation was attenuated by co-expression of hBMAL1a in a dose-dependent manner (Fig. 5a; Supplementary Fig. S5). To examine the effect of hBMAL1a on circadian rhythmic expression of clock gene, transcriptional activation of *hPer2-dsLuc* was monitored in NIH-3T3 cell line in real time for 72 h in the absence or presence of hBMAL1 isoforms. As shown in Fig. 5b, both the amplitude and period of the oscillatory expression of the *hPer2* promoter was drastically reduced by hBMAL1a (red line), but not by hBMAL1b (blue line). Interestingly, co-expression of hBMAL1a and hBMAL1b exerted a greater inhibitory effect on the amplitude of *hPer2* expression (Fig. 5b, green line).

To further evaluate the effect of hBMAL1a as a negative regulator of the molecular circadian clock, we generated a HeLa cell line in which expression of *hBmal1a* was specifically abolished by CRISPR/Cas9-mediated knockout while *hBmal1b* expression was intact (Supplementary Fig. S6a–e). Upon application of 50% horse serum for 2 h, the expression profiles of *hPer2*, *hBmal1b*, and *hBmal1a* mRNAs were assessed by qPCR for 48 h at 4 h intervals in normal or *hBmal1a* knockout (HeLa^*Bmal1a−/−*^) HeLa cells. As shown in Fig. 5c, d, the amplitude of *hBmal1b* and *hPer2* expression was increased in HeLa^*Bmal1a−/−*^ cells compared to normal HeLa cells. All together, these data offer the possibility that hBMAL1a might function as a negative regulator in the control of the human molecular clock.

## Discussion

Here, we report evidence that the human-specific isoform of BMAL1 may function as a negative regulator of the molecular circadian clock. BMAL1, along with its binding partner CLOCK, is an indispensable core clock component that is required for the transcriptional activation of downstream clock genes. Moreover, as shown in our previous studies, we have reported that the nuclear entry of the CLOCK:BMAL1 heterodimer is solely dependent on the NLS of BMAL1^21,22^. Thus, we reasonably presumed that the NLS-deficient hBMAL1a might exert a negative effect on the transcriptional activation mediated by canonical hBMAL1b and hCLOCK complex. Indeed, hBMAL1a effectively inhibited the transcriptional activation of *hPer1* promoter in a luciferase reporter assay (Fig. 5a; Supplementary Fig. S5). Furthermore, the amplitude of the oscillatory expression of *hPer2* was suppressed in the presence of hBMAL1a (Fig. 5b).

Based on our results, hBMAL1a may directly interact with hCLOCK or hBMAL1b in the course of inhibiting the transcriptional activation of downstream clock genes. The electrophoretic mobility of CLOCK protein differs depending on its phosphorylation status^29,30^. In mice, CLOCK mainly exists as a hypo-phosphorylated form throughout the day, while hyper-phosphorylated CLOCK is detected only at the phase of repression^29^. The level of the unphosphorylated CLOCK protein begins to decrease at the time of the transcriptional activation of the downstream clock gene *Dbp*^29^. Based on these previous observations, it is presumed that the upper and lower bands of human CLOCK in the present study represented the hypo- and unphosphorylated forms, respectively (Fig. 2b). First of all, hypo-phosphorylated hCLOCK is predominantly located in the nucleus, while both un- and hypo-phosphorylated forms exist in the cytoplasm (Fig. 2c, first and fourth lanes). As shown in Fig. 2b, hBMAL1b promotes an upward shift of the unphosphorylated form of hCLOCK (Fig. 2b, lanes 5–7). Simultaneously, nuclear entry of the hypo-phosphorylated form of hCLOCK was increased in the presence of exogenously expressed hBMAL1b (Fig. 2c, sixth lane). On the other hand, overexpression of hBMAL1a resulted in both the cytoplasmic retention of hCLOCK and an increase in the level of the unphosphorylated form of hCLOCK (Fig. 2a, b, lanes 5–7). These data suggest that hBMAL1b promotes both the phosphorylation and nuclear entry of hCLOCK (Fig. 5e, top half), while hBMAL1a prevents hCLOCK phosphorylation and nuclear translocation (Fig. 5e, bottom half).

Unexpectedly, we detected the unphosphorylated form of hCLOCK in the nuclear fraction in the presence of overexpressed hBMAL1a (Fig. 2c, fifth lane). As revealed by the BiFC assay, hBMAL1a was delivered into the nucleus by hBMAL1b (Fig. 3a). Moreover, hBMAL1a promoted the degradation of hBMAL1b in both the nucleus and cytoplasm (Fig. 3b; Supplementary Fig. S4a). Once delivered into the nucleus by hBMAL1b, hBMAL1a may dissociate from the complex as soon as it promotes the degradation of bound hBMAL1b. The free nuclear hBMAL1a then competes with its canonical isoform, hBMAL1b, for binding to hCLOCK in the nucleus. Actually, a portion of hCLOCK:hBMAL1a dimers was observed in the nuclei in the BiFC assay (Fig. 2a, top panels). Therefore, hBMAL1a might be able to promote the dephosphorylation of hCLOCK through a direct interaction in the nucleus.

A *hBmal1a*-specific knockout HeLa cell line was established to evaluate the physiological role of hBMAL1a. In the present study, rhythmic expression profiles of *hPer2*, *hBmal1b*, and *hBmal1a* mRNAs in HeLa cells were not as robust as in the normal human fibroblasts (Figs. 5c and 1c). However, the rhythmicity of *hPer2* and *hBmal1b* mRNA expression was somewhat restored in the *hBmal1a* knockout HeLa cells (Fig. 5d). Together, the data presented in this study indicate that hBMAL1a functions as a dominant-negative regulator of the molecular circadian clock. Studies of the dominant-negative effects of the splice variants of the transcription factors have been reported previously. For example, one of the isoforms of AFX (Foxo4), a member of the forkhead transcription factor family, is known to suppress the transcriptional activation of downstream genes by its canonical isoform in a dose-dependent manner^33^. The same pattern of dominant-negative regulation of transcription was reported for the transcription factors STAT3 or SND1^34,35^.

In conclusion, we are the first to show that a splice variant of human BMAL1 functions as a dominant-negative regulator of transcription. Interestingly, despite the similarity in the genomic sequence around exon 6 of the *Bmal1* gene in humans (exon 5–intron–exon 6–intron–exon 7) and mice (exon 5–intron–exon 6), the alternative splicing of exon 6 is observed only in humans (Fig. 1a). According to recent genomics studies, alternative RNA splicing patterns are not generally conserved in the human and mouse genomes, while most of the exons are strongly conserved in both genomes^36^. Taken together, we propose that the human-specific isoform of BMAL1 might be responsible for another level of fine-tuning in the control of human molecular circadian clock.

## Electronic supplementary material

Supplementary Figure S1, Supplementary Figure S2, Supplementary Figure S3, Supplementary Figure S4, Supplementary Figure S5, Supplementary Figure S6
