# Supplementary material for: A splice variant of human Bmal1 acts as a negative regulator of the molecular circadian clock
Source: Exp Mol Med. 2018 Dec 6;50(12):159. doi: 10.1038/s12276-018-0187-x (PMC6283877; doi:10.1038/s12276-018-0187-x)
Supplement: Supplementary file 1 — Supplementary Figure S1, Supplementary Figure S2, Supplementary Figure S3, Supplementary Figure S4, Supplementary Figure S5, Supplementary Figure S6 [file 12276_2018_187_MOESM1_ESM.pdf]

Supplementary Figure 1

a

```
Human Bmal1a     --------------------------------------------MINI-------ESMDTD
Human Bmal1b     MADQRMDISSTISDFMSPGPTDLLSSSLGTSGVDCNRKRKGSSTDYQ-------ESMDTD
Human Bmal1c     MADQRMDISSTISDFMSPGPTDLLSSSLGTSGVDCNRKRKGSSTDYQ-------ESMDTD
Human Bmal1d     MADQRMDISSTISDFMSPGPTDLLSSSLGTSGVDCNRKRKGSSTDYQ-------ESMDTD
Human Bmal1e     MADQRMDISSTISDFMSPGPTDLLSSSLGTSGVDCNRKRKGSSTDYQ-------ESMDTD
Human Bmal1f     MADQRMDISSTISDFMSPGPTDLLSSSLGTSGVDCNRKRKGSSTDYQ-------ESMDTD
Mouse Bmal1b     MADQRMDISSTISDFMSPGPTDLLSGSLGTSGVDCNRKRKGSATDYQ-------ESMDTD
Mouse Bmal1b’    MADQRMDISSTISDFMSPGPTDLLSGSLGTSGVDCNRKRKGSATDYQLDDFAFEESMDTD
Mouse Bmal1 i3   MADQRMDISSTISDFMSPGPTDLLSGSLGTSGVDCNRKRKGSATDYQL-----------
Mouse Bmal1 i4   MADQRMDISSTISDFMSPGPTDLLSGSLGTSGVDCNRKRKGSATDYQ-------ESMDTD
Mouse Bmal1 i5   MADQRMDISSTISDFMSPGPTDLLSGSLGTSGVDCNRKRKGSATDYQ-------ESMDTD
  Rat Bmal1 i1   MADQRMDISSTISDFMSPGPTDLLSGSLSTSGVDCNRKRKGSATDYQ-------ESMDTD
  Rat Bmal1 i2   MADQRMDISSTISDFMSPGPTDLLSGSLSTSGVDCNRKRKGSATDYQ-------ESMDTD
  Rat Bmal1 i3   MADQRMDISSTISDFMSPGPTDLLSGSLSTSGVDCNRKRKGSATDYQ-------ESMDTD
```

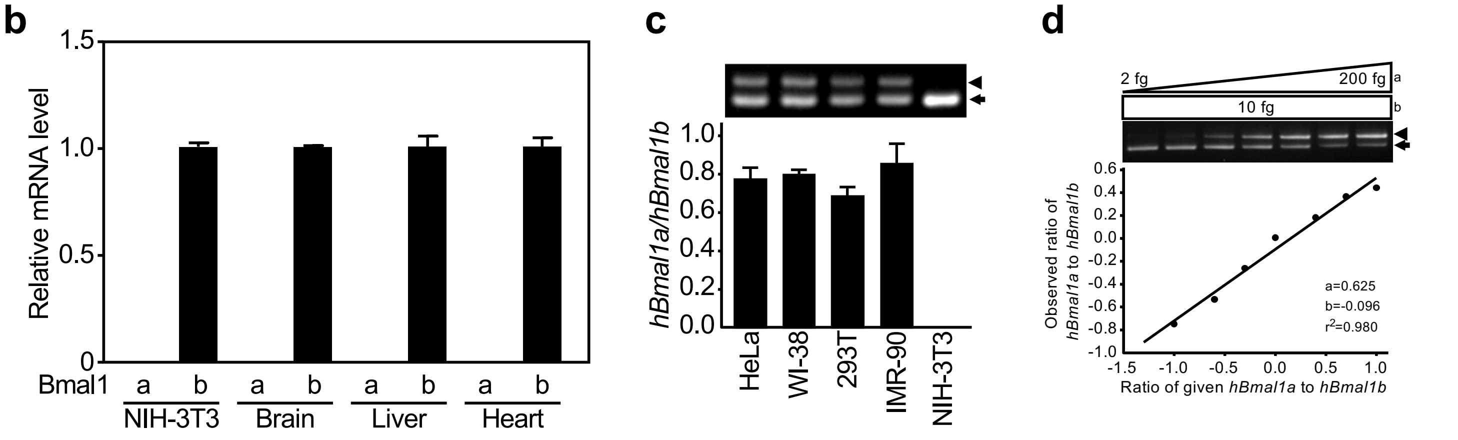

Supplementary Fig. S1 **a** Sequence alignment of the BMAL1 isoforms of human, mouse and rat. **b** Expression levels of *hBmal1a* or *hBmal1b* mRNA were analyzed by qPCR in mouse cell line and tissues. **c** Expression levels of *hBmal1a* (arrowhead) or *hBmal1b* (arrow) mRNA was analyzed by RT-PCR in human cell lines (HeLa, WI-38, HEK293T, and IMR-90) or mouse NIH-3T3 cells. **d** Titration and standard curve of competitive RT-PCR. A standard curve to quantify the ratio of *hBmal1a* to *hBmal1b* was constructed in the presence of 10 fg of hBmal1b cDNA and serial dilutions of the hBmal1a cDNA fragment (2 to 200 fg). The arrowhead and arrow indicate *hBmal1a* and *hBmal1b*, respectively.

Supplementary Figure 2

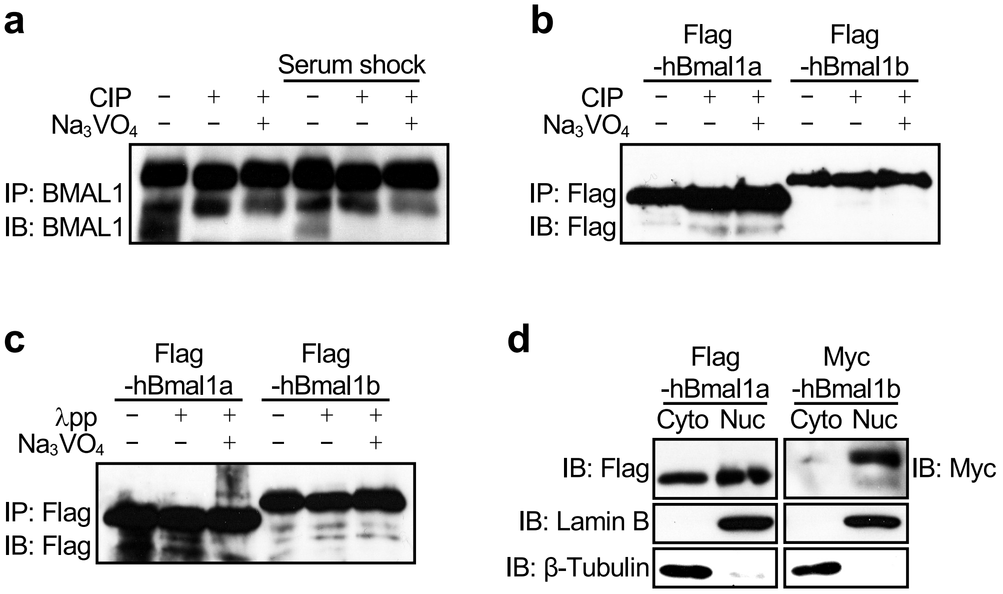

Supplementary Fig. S2 **a** Cell lysates obtained from HeLa cells treated with 50% horse serum or not was incubated with the calf intestinal phosphatase (CIP) in the presence or absence of the phosphatase inhibitor, sodium orthovanadate (Na_3VO_4). **b, c** CIP or lambda protein phosphatase (**λ**pp) were applied to HeLa cells transfected with Flag-hBmal1a or Flag-hBmal1b in the presence or absence of Na_3VO_4. **d** HeLa cells transfected with Flag-hBmal1a or Myc-hBmal1b were subjected to the nucleocytoplasmic fractionation. Western blotting was followed to investigate the expression levels of the proteins.

Supplementary Figure 3

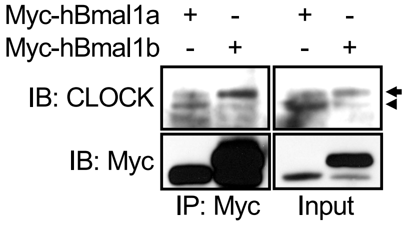

Supplementary Fig. S3 Interaction between exogenously expressed Myc-tagged hBMAL1 isoforms and the endogenous hCLOCK was analyzed by pull down assay using anti-Myc antibody. Arrow and arrowhead indicate the upper and lower hCLOCK bands, respectively.

Supplementary Figure 4

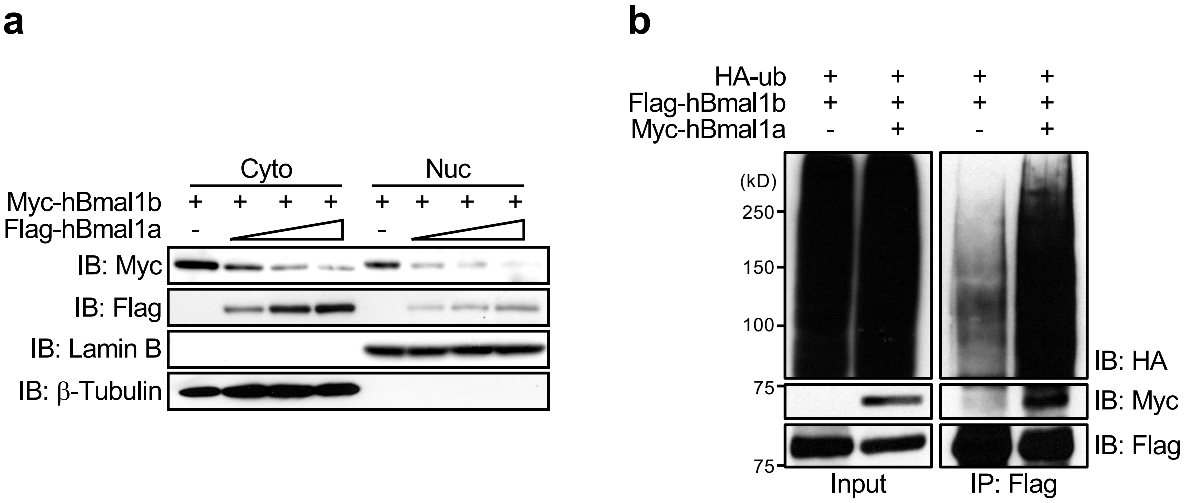

Supplementary Fig. S4 **a** HeLa cells transfected with a fixed amount of Myc-hBmal1b and increasing amounts of Flag-hBmal1a were subjected to nucleocytoplasmic fractionation. The expression levels of transfected proteins were detected by Western blotting using indicated antibodies. **b** HA-ub, Flag-hBmal1b and Myc-hBmal1a were transfected into HeLa cells as indicated. Cells were subjected to immunoprecipitation using anti-Flag antibody after treatment with 25 μM MG132 for 5 hours.

Supplementary Figure 5

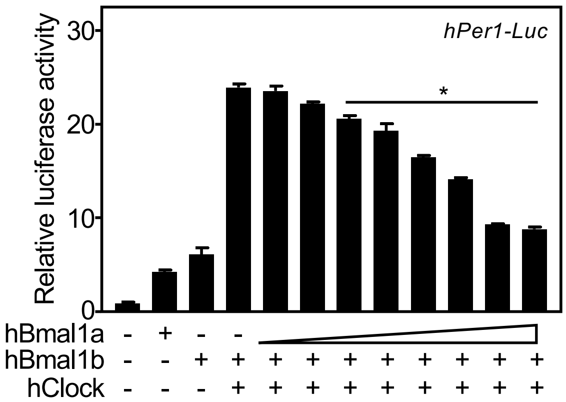

Supplementary Fig. S5 Transcriptional activation of the *hPer1*-Luciferase reporter by hCLOCK:hBMAL1b was decreased in the presence of hBMAL1a in a dose-dependent manner. The bioluminescence was measured at 48 hours after transfection. The resulting luciferase activities were shown as the means ± S.E. (n = 3) relative to the control group (* $P < 0.01$).

Supplementary Figure 6

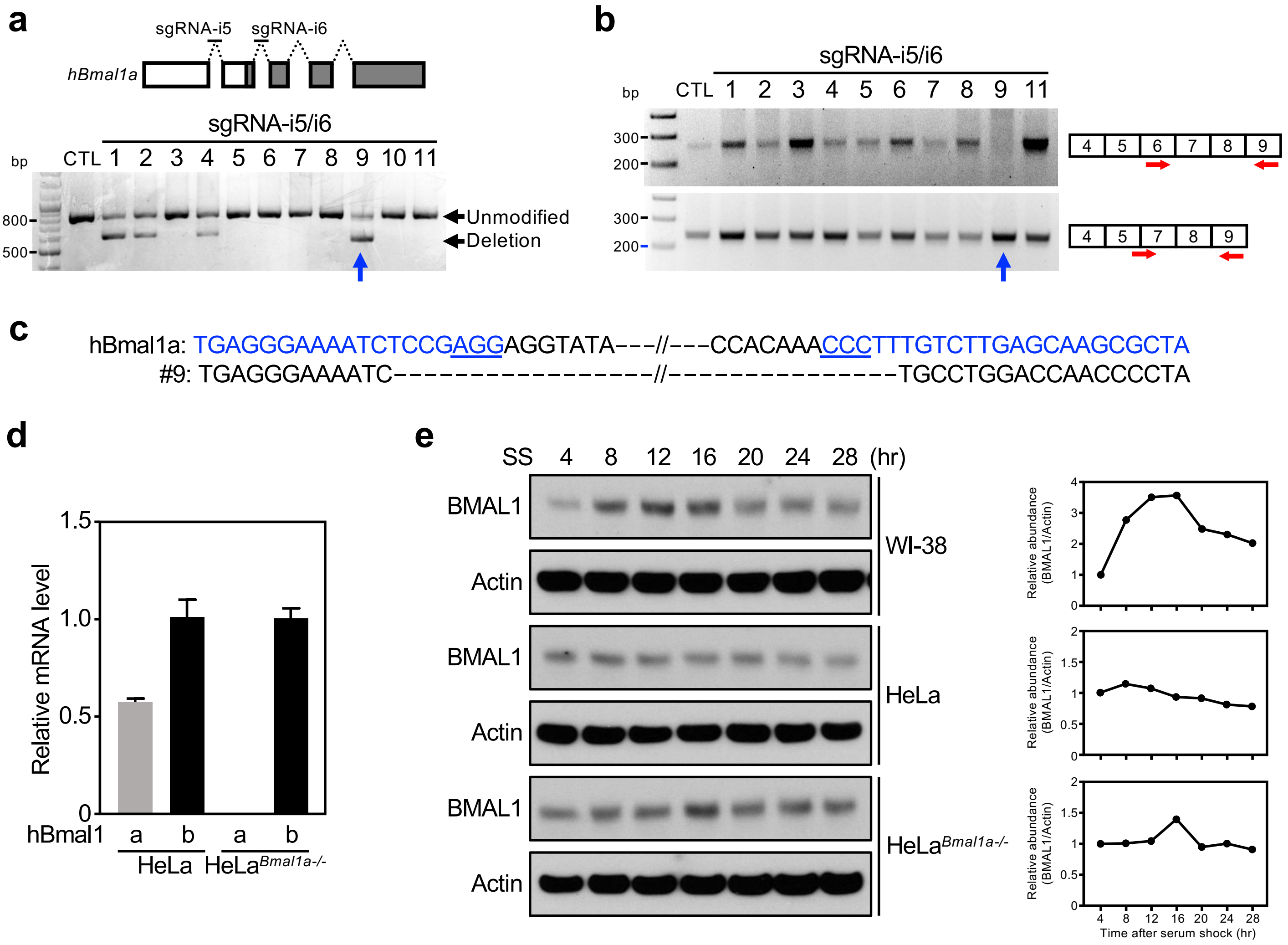

Supplementary Fig. S6 CRISPR/Cas9-mediated knockout of *hBmal1a* in HeLa cells. **a** Guide RNAs targeting intron 5 (sgRNA-i5) and intron 6 (sgRNA-i6) were generated, validated by T7E1 assay, and transfected into HeLa cells. After selection against Puromycin, total 11 stable cell lines were generated. PCR assay was performed using genomic DNAs purified from the control and selected cell lines to validate the deletion of exon 6. **b** Deletion of exon 6 was further validated by RT-PCR. By genomic and RT-PCRs, the #9 stable cell line (blue arrow) turned out to be the positive cell line for exon 6 knockout. **c** By sequencing on the PCR bands obtained from the genomic PCR, it was confirmed that 247 bp including exon 6 was missing from the *hBmal1a* knockout cell line (blue characters indicate the sequence of guide RNAs). **d** Knockout of *hBmal1a* was further validated by qPCR in HeLa or HeLa$^{Bmal1a-/-}$ cells. **e** Circadian expression profiles of BMAL1 protein in WI-38, HeLa or HeLa$^{Bmal1a-/-}$ cell lines. Upon application of 50% horse serum for 2 hours, Bmal1 protein levels were analyzed by Western blotting up to 28 hours at 4 hours of intervals.
